# Regenerative cell therapy for pulmonary arterial hypertension in animal models: a systematic review

**DOI:** 10.1186/s13287-019-1172-6

**Published:** 2019-03-06

**Authors:** Colin M. Suen, Duncan J. Stewart, Joshua Montroy, Christopher Welsh, Brendan Levac, Neil Wesch, Alexander Zhai, Dean Fergusson, Lauralyn McIntyre, Manoj M. Lalu

**Affiliations:** 1 Regenerative Medicine Program, The Ottawa Hospital Research Institute, 501 Smyth Road, PO Box 201B, Ottawa, ON K1H 8L6 Canada; 2 Department of Cell and Molecular Medicine, University of Ottawa, Ottawa, Canada; 3 Department of Anesthesiology and Pain Medicine, The Ottawa Hospital, The Ottawa Hospital Research Institute, Ottawa, Canada; 4 Clinical Epidemiology Program, Ottawa, Canada; 5 Blueprint Translational Research Group, The Ottawa Hospital Research Institute, Ottawa, Canada; 6 Department of Medicine, University of Ottawa, Ottawa, Canada; 7 Depatrment of Surgery, University of Ottawa, Ottawa, Canada; 8 Department of Epidemiology and Community Medicine, University of Ottawa, Ottawa, Canada

**Keywords:** Meta-analysis, Pulmonary hypertension, Cell therapy, Animal models of human disease

## Abstract

**Background:**

Pulmonary arterial hypertension (PAH) is a rare disease characterized by widespread loss of the pulmonary microcirculation and elevated pulmonary arterial pressures leading to pathological right ventricular remodeling and ultimately right heart failure. Regenerative cell therapies could potentially restore the effective lung microcirculation and provide a curative therapy for PAH. The objective of this systematic review was to compare the efficacy of regenerative cell therapies in preclinical models of PAH.

**Methods:**

A systematic search strategy was developed and executed. We included preclinical animal studies using regenerative cell therapy in experimental models of PAH. Primary outcomes were right ventricular systolic pressure (RVSP) and mean pulmonary arterial pressure (mPAP). The secondary outcome was right ventricle/left ventricle + septum weight ratio (RV/LV+S). Pooled effect sizes were undertaken using random effects inverse variance models. Risk of bias and publication bias were assessed.

**Results:**

The systematic search yielded 1285 studies, of which 44 met eligibility criteria. Treatment with regenerative cell therapy was associated with decreased RVSP (SMD − 2.10; 95% CI − 2.59 to − 1.60), mPAP (SMD − 2.16; 95% CI − 2.97 to − 1.35), and RV/LV+S (SMD − 1.31, 95% CI − 1.64 to − 0.97). Subgroup analysis demonstrated that cell modification resulted in greater reduction in RVSP. The effects on RVSP and mPAP remained statistically significant even after adjustment for publication bias. The majority of studies had an unclear risk of bias.

**Conclusions:**

Preclinical studies of regenerative cell therapy demonstrated efficacy in animal models of PAH; however, future studies should consider incorporating design elements to reduce the risk of bias.

**Systematic review registration:**

Suen CM, Zhai A, Lalu MM, Welsh C, Levac BM, Fergusson D, McIntyre L and Stewart DJ. Efficacy and safety of regenerative cell therapy for pulmonary arterial hypertension in animal models: a preclinical systematic review protocol. *Syst Rev*. 2016;5:89.

**Trial registration:**

CAMARADES-NC3Rs Preclinical Systematic Review & Meta-analysis Facility (SyRF). http://syrf.org.uk/protocols/. Syst Rev 5:89, 2016

**Electronic supplementary material:**

The online version of this article (10.1186/s13287-019-1172-6) contains supplementary material, which is available to authorized users.

## Background

Pulmonary arterial hypertension (PAH) is a progressive disease associated with increased pulmonary vasculature resistance, increased pulmonary arterial pressure, and right heart failure [1]. The clinical diagnosis of pulmonary arterial hypertension (PAH) is defined by a mean pulmonary arterial pressure ≥ 25 mmHg at rest and pulmonary capillary wedge pressure ≤ 15 mmHg by right heart catheterization [2]. Although the mechanisms leading to PAH are still unclear, endothelial apoptosis is widely considered to be an initiating process that reduces the effective lung vasculature area through functional pulmonary microvascular rarefaction and obliterative remodeling of the small pulmonary arterioles due to the emergence of growth dysregulated vascular cells and endothelial cell dropout [3]. Ultimately, loss of lung microcirculation leads to progressive increases in pulmonary vascular resistance, right ventricular remodeling, and eventually right heart failure [2, 4, 5].

Regenerative cell therapy has emerged as a novel treatment for PAH that has been examined in many preclinical animal studies and applied in several clinical trials [6, 7]. The majority of preclinical models report the use of two main cell types: early-outgrowth endothelial progenitor cells (EPCs, also known as circulating angiogenic cells, myeloid angiogenic cells) and mesenchymal stromal cells (MSCs, also known as mesenchymal stem cells, adult stem cells) [6, 8]. EPCs and MSCs have demonstrated the ability to migrate to sites of vascular injury in several in vivo animal disease models [9] secreting paracrine factors which induce vascular repair and reduce inflammation [6]. Preclinical studies involving EPCs and MSCs have demonstrated efficacy by reducing pulmonary pressures, regenerating lost microvascular area, and reducing both pulmonary vascular and right ventricular remodeling [6]. Three small clinical trials (2 adult, 1 pediatric) involving regenerative cell therapy on PAH patients have been completed and demonstrate some promise in limiting disease burden [7, 10, 11]. However, to date, there has been no systematic synthesis of preclinical studies investigating stem cell therapy for the treatment of PAH. A synthesis of this preclinical data may identify knowledge gaps, impact the design of further preclinical testing of cell therapies, and potentially influence the design of future clinical trials.

In this systematic review and meta-analysis, we quantified the effects of regenerative cell therapy on pulmonary hemodynamics based on currently available data in preclinical studies. Cells from all sources were considered, as well as all enhancements (e.g., gene transfected). We also provide a comprehensive review of study methodology, assessment of bias, and publication bias.

## Methods/design

### Protocol and registration

Our protocol was published [12] and also posted on the Collaborative Approach to Meta Analyses and Review of Animal Data from Experimental Studies (CAMRADES) website (http://syrf.org.uk). Reporting of this review adheres to the Preferred Reporting Items for Systematic Reviews and Meta-Analyses guidelines (Additional file 1) [13].

### Eligibility criteria

We included interventional studies (randomized, pseudo-randomized, and non-randomized) that examined an in vivo model of experimentally induced PAH comparing the effect of regenerative cell administration versus a diseased control. Both unmodified cells and enhanced cells (e.g., gene transfected) were considered. Valid preclinical in vivo models of PAH that reproduced features of the pathophysiology associated and/or etiology of human PAH [1] were the rodent monocrotaline (MCT) and SU5416 + chronic hypoxia (SU+CH) models. Prevention and treatment type studies were included. Mouse models were excluded in this systematic review as available models at the time of data extraction (chronic hypoxia) lacked significantly elevated pulmonary pressures, right ventricular hypertrophy, and pulmonary arteriolar remodeling [14]. Animal models of PAH secondary to other causes such as left heart disease, lung disease, or thromboembolism (WHO groups 3–5) [2] were excluded. Genetically modified animals and neonatal animal models were also excluded.

Outcomes were assessed at least 1 week after intervention to exclude the possibility of acute effects of cell administration. Our primary outcomes were measures of pulmonary hemodynamics (mean pulmonary arterial pressure, right ventricular systolic pressure). Secondary outcomes included survival and right ventricular (RV) remodeling expressed as the weight ratio of right ventricle/left ventricle + septum (RV/LV+S).

### Literature search

We searched Ovid MEDLINE®, Ovid MEDLINE® In-Process & Other Non-Indexed Citations, and EMBASE Classic+ until January 2018. The search strategy was developed by an information specialist and validated using the Peer Review of Electronic Search Strategies (PRESS) by another information specialist (Additional file 2) [15]. The search used combination of controlled vocabulary (for example stem cells, pulmonary hypertension), and keywords (for example EPC, MSC, iPSC, HSC, PAH) and parsing were formatted accordingly to each database. In addition, we performed a manual review of the bibliographies of selected articles and relevant reviews. Only articles in the English language were included in the review.

### Study selection process

**Fig. 1:**
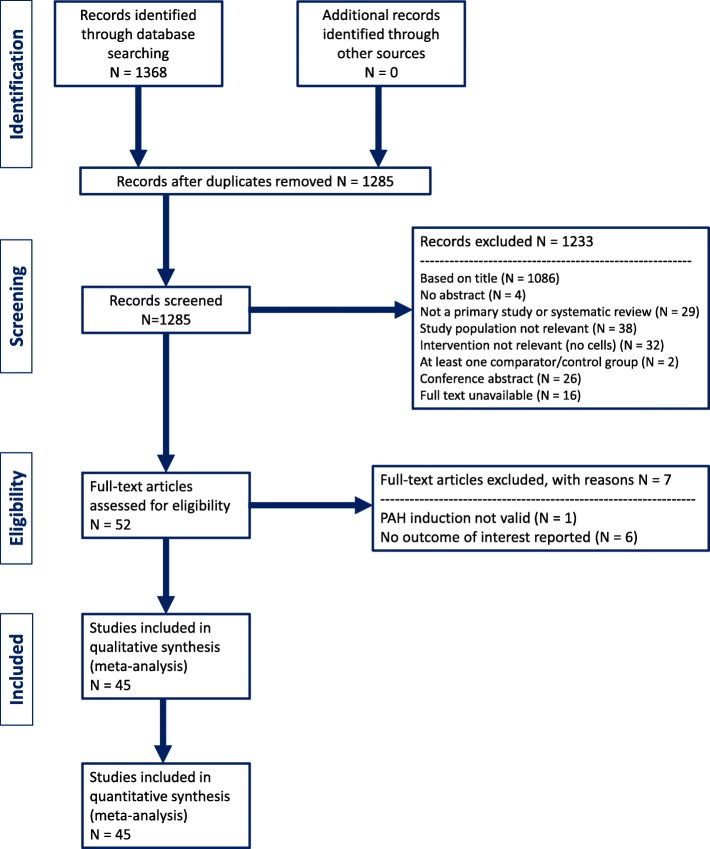
PRISMA flow diagram

Citations from the literature search were collated, and duplicate studies removed. Titles and abstracts of search results were screened independently by two reviewers (CW, BL, NW, CS) using DistillerSR software (Evidence Partners, Ottawa, ON). Abstracts deemed potentially relevant were recorded, and full-text articles obtained. Remaining articles moved forward for full-text review, which was also performed in duplicate (CW, BL, NW, CS). Disagreements between reviewers were resolved by consensus or by a third-party consultation (DJS or MML). The study selection process was documented and reported using a PRISMA flow diagram (Fig. 1) [13].

### Data extraction and risk of bias assessment

Data was extracted independently by two individuals into standardized, electronic pilot-tested forms. Data was collected on general study characteristics (e.g., study design, funding source, and origin), on regenerative cell characteristics (cell type, dose, enhancements, etc.), and on primary and secondary outcomes. Disagreements during extraction were resolved by consensus or third-party consultation (MML or DJS). In the case of missing or unclear data for the primary or secondary outcome measures, an attempt was made to contact the primary study author(s) for clarification. Risk of bias was assessed independently in duplicate for each included study using the SYRCLE (Systematic Review Centre for Laboratory animal Experimentation) risk of bias tool [16], and each parameter for the type of bias was scored as low, high, or unclear risk of bias.

### Data analysis

Studies were pooled using Comprehensive Meta-Analyst (v3; Biostat Inc., USA). For continuous endpoints, mean difference (MD) or standardized mean difference (SMD) was calculated, depending on the measurement of the outcome. MD and SMD were calculated using random effects of inverse variance meta-analyses and presented with accompanying 95% confidence intervals. For dichotomous outcomes, risk ratios were calculated using a random effects analysis based on the Der-Simonian Laird model and presented with accompanying 95% confidence intervals. Statistical heterogeneity of included studies was measured using the *I*^*2*^ statistic [17]. An *I*^*2*^ value of > 50% was determined to indicate important heterogeneity worth further exploration. We assessed the potential for publication bias using funnel plots and Egger’s regression test [18].

A priori defined subgroup analyses were examined on the primary endpoint of right heart catheterization hemodynamics (RVSP/mPAP). The pre-planned subgroups that were analyzed included regenerative cell type, cell enhancement (cell pretreatments/priming, gene transfection), and timing of administration. For timing of cell therapy, interventions administered prior to 14 days were considered “early” based on the rat monocrotaline model (where hemodynamic changes are not noted prior to this timepoint after disease induction) [14]. A post hoc subgroup analysis was performed examing the effect of cell compatibility (i.e., allogeneic and xenogeneic) on the primary endpoint of right heart catheterization hemodynamics (RVSP/mPAP). In addition, we performed post hoc subgroup analysis exploring the effect of cell dose and cell origin (bone marrow, umbilical, adipose) on studies which administered MSCs.

## Results

### Study characteristics

Our systematic search yielded a total of 1285 articles. After preliminary screening, 94 articles were identified for full-text review, of which 45 studies met eligibility criteria for this review (Fig. 1). Baseline characteristics are reported in Table 1. Studies were published between 2003 and 2017, with 14 studies from China, 8 studies from the USA, 6 studies from Korea, 6 studies from Japan, 5 from Taiwan, 2 from Canada, and 1 each from Netherlands, Brazil, Egypt, and Italy. Sample size ranged from 7 to 51, and follow-up duration ranged from 7 to 173 days. The majority of the studies were conducted in rats (43 out of 45), with 2 studies in dogs [19, 20].

**Table 1:** Summary of study characteristics of included studies

		Animal model characteristics			Cell characteristics						
Reference	Country	Species (strain)	Intervention (days)	Follow-up (days)	Stem cell type	Cell species of origin	Tissue of origin for cell product	Transplant type	Dose	Route	Cell enhancement
Nagaya 2003 [32]	Japan	Rat (nude athymic)	7	14	EPC	Human	Umbilical cord blood	Xenogenic	1,000,000	IV (intrajugular)	Adrenomedullin transfection
Takahashi 2004 [19]	Japan	Dog (beagle)	14	28	EPC	Dog	Peripheral blood	Autologous	1,000,000	IV (intrajugular)	
Zhao 2005a [33]	Canada	Rat (Fischer)	21	14	EPC	Rat	Bone marrow	Allogeneic	1,000,000	IV (intrajugular)	eNOS
Zhao 2005b [33]	Canada	Rat (Fischer)	3	21	EPC	Rat	Bone marrow	Allogeneic	1,500,000	IV (intrajugular)	
Kanki-Horimoto 2006 [34]	Japan	Rat (Sprague Dawley)	7	14	MSC	Rat	Bone marrow	Allogeneic	1,000,000	IV (intrajugular)	
Baber 2007 [35]	USA	Rat (Sprague Dawley)	14	35	MSC	Rat	Bone marrow	Allogeneic	3,000,000	Intratracheal	
Spees 2008	USA	Rat (Sprague Dawley)	21	21	BM-MNC	Rat	Bone marrow	Allogeneic	5,000,000	IV (tail vein)	
Yip 2008a [36]	Taiwan	Rat (Sprague Dawley)	7	83	EPC	Rat	Bone marrow	Autologous	1,200,000	IV (tail vein)	
Yip 2008b [36]	Taiwan	Rat (Sprague Dawley)	7	21	EPC	Rat	Bone marrow	Autologous	1,200,000	IV (tail vein)	
Sun 2009a [37]	Taiwan	Rat (Sprague Dawley)	3	42	EPC	Rat	Bone marrow	Autologous	2,000,000	IV (tail vein)	Cilostazol pretreatment
Sun 2009b [37]	Taiwan	Rat (Sprague Dawley)	3	42	EPC	Rat	Bone marrow	Autologous	2,000,000	IV (tail vein)	
Umar 2009 [38]	Netherlands	Rat (Wistar)	14	14	MSC	Rat	Bone marrow	Allogeneic	1,000,000	IV (intrajugular)	
Ormiston 2010 [22]	Canada	Rat (nude athymic)	3	21	EPC (early and late)	Human	Peripheral blood	Xenogenic	1,500,000	IV (intrajugular)	
Takemiya 2010 [39]	Japan	Rat (Lewis)	14	28	MSC	Rat	Bone marrow	Allogeneic	500,000	IV (tail vein)	PCS transfection
Angelini 2011 [40]	Italy	Rat (Sprague Dawley)	28	7	MSC	Rat	Adipose	Allogeneic	4,000,000	IV (tail vein)	
Mirsky 2011a [41]	USA	Rat (nude athymic)	35	10 and 15	EPC	Human	Peripheral blood	Xenogenic	1,500,000	IV (tail vein)	
Mirsky 2011b [41]	USA	Rat (nude athymic)	35	10 and 15	EPC	Human	Peripheral blood	Xenogenic	1,500,000	IV (tail vein)	
Jiang 2012 [42]	China	Rat (Sprague Dawley)	3	18	MSC		Bone marrow	Allogeneic	4,000,000	IV (tail vein)	
Kim 2012 [43]	Korea	Rat (Sprague Dawley)	7	28	BM-MNC	Rat	Bone marrow	Allogeneic	20,000,000	IV (tail vein)	
Luan 2012 [30]	China	Rat (Sprague Dawley)	21	14	MSC	Rat	Bone marrow	Allogeneic	100,000	IV (right femoral vein)	
Luan 2012 [20]	China	Dog (Mongrel)	14	56	BM-MNC	Dog	Bone marrow	Allogeneic	1,300,000	Intratracheal	
Sun 2012 [44]	Taiwan	Rat (Sprague Dawley)	3	35	EPC	Rat	Bone marrow	Autologous	2,000,000	IV (tail vein)	
Xie 2012 [45]	China	Rat (Sprague Dawley)	7	14	MSC	Rat	Bone marrow	Allogeneic	1,000,000	IV (right femoral vein)	
Yen 2013 [46]	Taiwan	Rat (Sprague Dawley)	21	69	EPC	Rat	Bone marrow	Autologous	2,000,000	IV (penile vein)	
Zhou 2013a [47]	USA	Rat (Fischer)	3	25	EPC	Rat	Bone marrow	Allogeneic	1,500,000	IV (intrajugular)	COX1-PCS transfection
Zhou 2013b [47]	USA	Rat (Fischer)	21	28	EPC	Rat	Bone marrow	Allogeneic	1,500,000	IV (intrajugular)	COX1-PCS transfection
Chen 2014 [48]	China	Rat (Sprague Dawley)	21	14	MSC	Rat	Bone marrow	Allogeneic	1,000,000	IV (tail vein)	
Eguchi 2014 [49]	Japan	Rat (Wistar)	7	21	MSC	Rat	Adipose	Allogeneic	7,000,000	IV (tail vein)	
Guo 2014 [50]	China	Rat (Sprague Dawley)	21	21	MSC	Rat	Bone marrow	Allogeneic	5,000,000	IV (intrajugular)	
Luan 2014 [51]	China	Rat (Sprague Dawley)	7	173	MSC	Rat	Bone marrow	Allogeneic	10,000,000	IV (sublingual vein)	
Somanna 2014 [52]	USA	Rat (Sprague Dawley)	14	14	MSC	Rat	Adipose	Allogeneic	3,000,000	Intratracheal	COX1 transfection
Ikutomi 2015a [53]	Japan	Rat (Fischer)	0, 1, 3, 5, 7, 9	19	EPC-early	Rat	Bone marrow	Allogeneic	1 × 10^6^ per injection (total 6×)	IV (tail vein)	
Ikutomi 2015b [53]	Japan	Rat (Fischer)	0, 1, 3, 5, 7, 9	19	EPC-late	Rat	Bone marrow	Allogeneic	1 × 10^6^ per injection (total 6×)	IV (tail vein)	
Ikutomi 2015c [53]	Japan	Rat (Fischer)	0, 1, 3, 5, 7, 9	19	EPC-very late	Rat	Bone marrow	Allogeneic	1 × 10^6^ per injection (total 6×)	IV (tail vein)	
Kang 2015 [54]	Korea	Rat (Lewis)	14	14	MSC	Human	Umbilical cord blood	Allogeneic	250,000	IV (tail vein)	S1p priming
Lee 2015 [55]	Korea	Rat (Sprague Dawley)	7	7	MSC	Human	Umbilical cord blood	Xenogenic	3,000,000	IV (intrajugular)	
Liang 2015 [56]	China	Rat (Sprague Dawley)	7 or 14	7	MSC	Rat	Adipose	Allogeneic	1,000,000	IV (intrajugular)	
Liu 2015 [57]	China	Rat (Sprague Dawley)	5	16	MSC	Human	Umbilical cord blood	Xenogenic	1,000,000	IV (tail vein)	
Luo 2015 [58]	China	Rat (Sprague Dawley)	14	7	MSC	Rat	Adipose	Allogeneic	1,000,000	IV (intrajugular)	
Pan 2015 [59]	China	Rat (Sprague Dawley)	7, 14, 21	7,14,21	EPC	Rat	Bone marrow	Allogeneic	1,000,000	IV (tail vein)	shRNA-CD40 transfection
Chen 2016a [60]	China	Rat (Wistar)	14	21	MSC	Rat	Bone marrow	Allogeneic	1,000,000	IV (tail vein)	eNOS/F92A-Cav1
Chen 2016b [60]	China	Rat (Wistar)	14	21	MSC	Rat	Bone marrow	Allogeneic	1,000,000	IV (tail vein)	F92A-Cav1
Chen 2016c [60]	China	Rat (Wistar)	14	21	MSC	Rat	Bone marrow	Allogeneic	1,000,000	IV (tail vein)	eNOS
Huang 2016 [61]	Taiwan	Rat (Sprague Dawley)	0 or 14	14	iPSC	Mouse	Not reported	Xenogenic	2,000,000	IV (tail vein)	None (compared to conditioned media)
Kim 2016 [62]	Korea	Rat (Sprague Dawley)	7	7 and 21	MSC	Human	Umbilical cord blood	Xenogenic	3 × 10^6^/ml/cm^2^	IV (intrajugular)	
Lim 2016 [63]	Korea	Rat (Lewis)	14	14	MSC	Human	Bone marrow	Xenogenic	250,000	IV (tail vein)	C1P (ceramide-1 phosphate) priming
Rathinasabapathy 2016 [64]	USA	Rat (Sprague Dawley)	14	14	MSC	Rat	Adipose	Allogeneic	1,000,000	IP	
Varshney 2016 [65]	USA	Rat (Sprague Dawley)	3	18	MSC	mouse	Not reported	Xenogenic	3,500,000	IV (intrajugular)	GFP-SKL (secreted Klotho) transfection
Ahmed 2017 [66]	Egypt	Rat (Wistar)	14	14	EPC	Rat	Bone marrow	Allogeneic	1,000,000	IV (no specific site indicated)	pinocembrin preconditioning
Cheng 2017 [67]	China	Rat (Lewis)	21	21	MSC	Rat	Bone marrow	Allogeneic	3,000,000	IV (tail vein)	Let7a transfection
de Mendonca 2017 [68]	Brazil	Rat (Wistar)	14	21	MSC	Rat	Adipose	Allogeneic	100,000	IV (intrajugular)	
Lee 2017 [69]	Korea	Rat (Sprague Dawley)	1, 7	14 and 28	MSC	Human	Umbilical cord blood	Xenogenic	3,000,000, 1,000,000, 300,000	IV (intrajugular) (multiple doses and timing)	
Middleton 2017 [70]	USA	Rat (Sprague Dawley)	14	21	CDC	Rat	Heart	Allogeneic	2,000,000	IV (intrajugular)	
Luo 2018 [71]	China	Rat (Sprague Dawley)	14	21	MSC	Rat	Adipose	Allogeneic	1,000,000	IV (intrajugular)	Adiponectin transfection

Letters following the author and year (ex. Chen 2016a) indicate that more than one experiment or treatment was conducted in the same publication

Studied cell types included bone marrow mononuclear cell (BM-MNC) (*n* = 3), cardiosphere-derived cells (CDC) (*n* = 1), endothelial progenitor cell (EPC) (*n* = 13), induced pluripotent stem cell (iPSC) (*n* = 1), and mesenchymal stem cell (MSC) (*n* = 27). Forty-one studies (91%) administered the cells intravenously, three studies (7%) administered the cells intratracheally, and one study (2%) administered the cell intra-peritoneally. The cell dose used ranged from 100,000 total cells to 20 million total cells. The timing of cell administration ranged from immediately following PAH induction to 35 days post-PAH induction. Seventeen studies used cell enhancement strategies, and 28 studies did not. All studies used a fresh cell product.

### Primary outcomes

#### Right ventricular systolic pressure (RVSP)

**Fig. 2:**
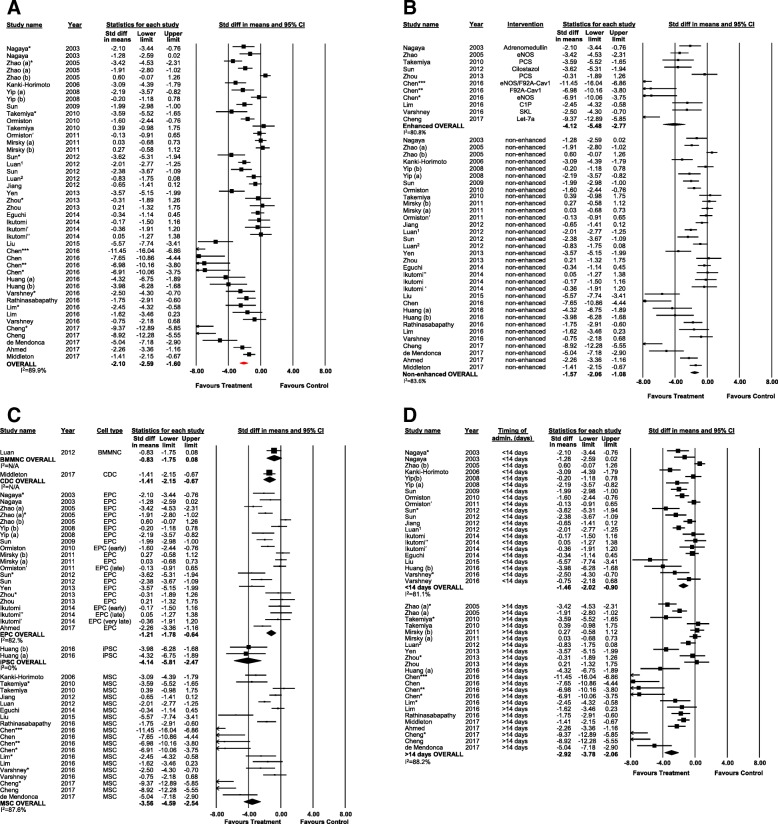
Forest plot of effect of regenerative cell therapy on RVSP in preclinical models of PAH (**a**). Reported in standardized mean difference and 95% confidence intervals. Subgroup analysis of RVSP and **b** cell enhancement, **c** cell type, and **d** timing of administration. Letters indicate two separate experiments within the same publication. Asterisk denotes cell enhancement within the same experiment, right single quotation mark indicates different cell subtype within the same experiment, and subscript number denotes a separate study/publication within the same year

Twenty-six studies reported data on RVSP from 44 experiments (805 animals). Cell therapy was associated with a significant reduction in RVSP compared to control (SMD − 2.10; 95% CI − 2.59 to − 1.60) (Fig. 2a). In a post hoc analysis using weighted mean difference (WMD), animals which had received regenerative cell therapy saw a 13.7-mmHg reduction in RVSP (95% CI − 16.2 to − 11.1) (Additional file 3: Figure S1).

The treatment effect was analyzed by pre-defined subgroups (cell type, cell enhancement, and timing of treatment, species). Cell enhancement by gene transfection or small molecule pretreatment/priming was associated with a greater reduction in RVSP compared to non-enhanced cells (Fig. 2b) (*p* = 0.001). Subgroup analysis by cell type showed significant reductions in RVSP for all cell types, with the exception of bone marrow mononuclear cells (Fig. 2c). For bone marrow mononuclear cells (BM-MNC) and cardiosphere-derived cells (CDC) cell types, only one study was available to contribute data to each. Post hoc analysis demonstrated that MSCs had a significantly greater effect in lowering RVSP than EPCs (*p* < 0.001). Cell therapy administered at 14 days or later was associated with a greater reduction in RVSP compared to cell therapy administered less than 14 post disease induction (Fig. 2d). In a post hoc subgroup analysis of cell compatibility, there was no difference in effect between allogeneic, autologous, or xenogeneic cell therapy (Additional file 3: Figure S2).

Visual inspection of the funnel plot indicated potential publication bias, which was supported by Egger’s regression (*p* < 0.001). Post hoc trim and fill analysis suggests a 45% overestimation of effect. Accounting for potential publication bias, cell therapy remained associated with a significant reduction in RVSP (SMD − 1.45; 95% CI − 1.98 to − 0.92) (Additional file 3: Figure S3). In post hoc analyses of MSC studies only, there was no difference in effect between high vs low dose, or MSC source (Additional file 3: Figures S4-S5).

### Mean pulmonary arterial pressure (mPAP)

**Fig. 3:**
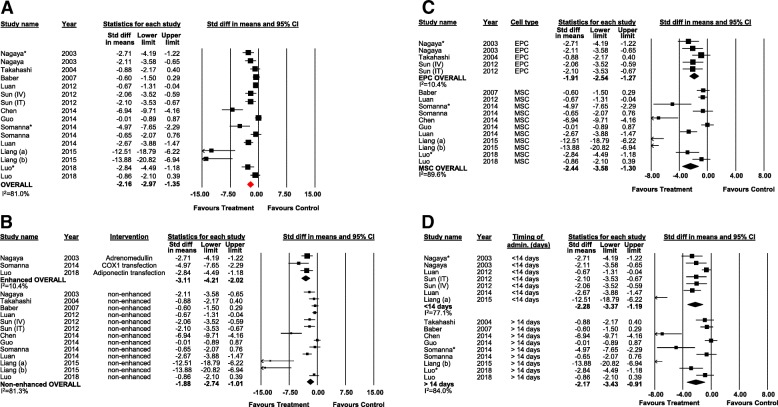
Forest plot of mean pulmonary arterial pressure in preclinical models of PAH. **a** mPAP for all studies. Subgroup analysis of mPAP by **b** enhancement, **c** cell type, and **d** timing of intervention. Reported in standardized mean difference and 95% confidence intervals. IV intravenous, IT intratracheal. Letters indicate two separate experiments within the same publication. Asterisk denotes cell enhancement within the same experiment

Eleven studies (16 experiments) reported data on mPAP (16 experiments, 233 animals). Overall, regenerative cell therapy was associated with a significant reduction in mPAP compared to control (SMD − 2.16; 95% CI − 2.97 to − 1.35) (Fig. 3a). There was no difference in effect between enhanced and non-enhanced cells, although data from enhanced cell studies was sparse (*n* = 3) (Fig. 3b). Subgroup analysis by cell type showed significant reductions in mPAP for treatment with EPC and MSC; however, post hoc analysis demonstrated no significant benefit of one cell type over the other (Fig. 3c). Subgroup analysis by timing of treatment suggests efficacy when cell therapy was administered at less than 21 days (Fig. 3d). In a post hoc subgroup analysis of cell compatibility, there was no difference in effect between allogeneic, autologous, or xenogeneic cell therapy (Additional file 3: Figure S6).

Visual inspection of the funnel plot indicated potential publication bias, which was confirmed by Egger’s regression (*p* < 0.001) (Additional file 3: Figure S7). Post hoc trim and fill analysis suggests a 5% overestimation of effect. Accounting for potential publication bias, cell therapy remained associated with a significant reduction in mPAP (SMD − 2.05; 95% CI − 2.90 to − 1.21) (Additional file 3: Figure S7). In post hoc analyses of MSC studies only, there was no difference in effect between different MSC sources; however, a larger effect was observed with a low dose of cells (< 1 million cells) (Additional file 3: Figures S8-S9).

### Secondary outcomes

**Fig. 4:**
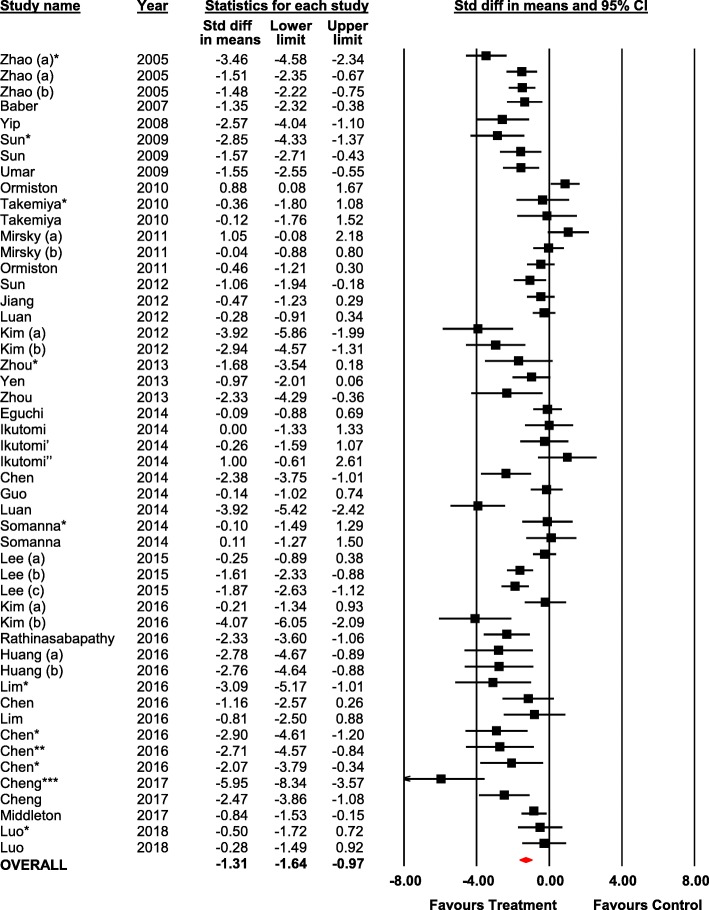
Forest plot of Fulton’s index (RV/LV+S ratio). Reported in standardized mean difference and 95% confidence intervals. Letters indicate two separate experiments within the same publication

Twenty-nine studies (50 experiments) reported RV/LV+S from 930 animals. Regenerative cell therapy was associated with an overall decrease in RV remodeling measured by RV/LV+S (SMD − 1.31, 95% CI − 1.64 to − 0.97) (Fig. 4). Three studies reported data on mortality (87 animals). No statistically significant difference in mortality was observed between groups (RR 2.11, 95% CI 0.12 to 36.04) (Additional file 3: Figure S10).

### Risk of bias (SYRCLE tool)

**Fig. 5:**
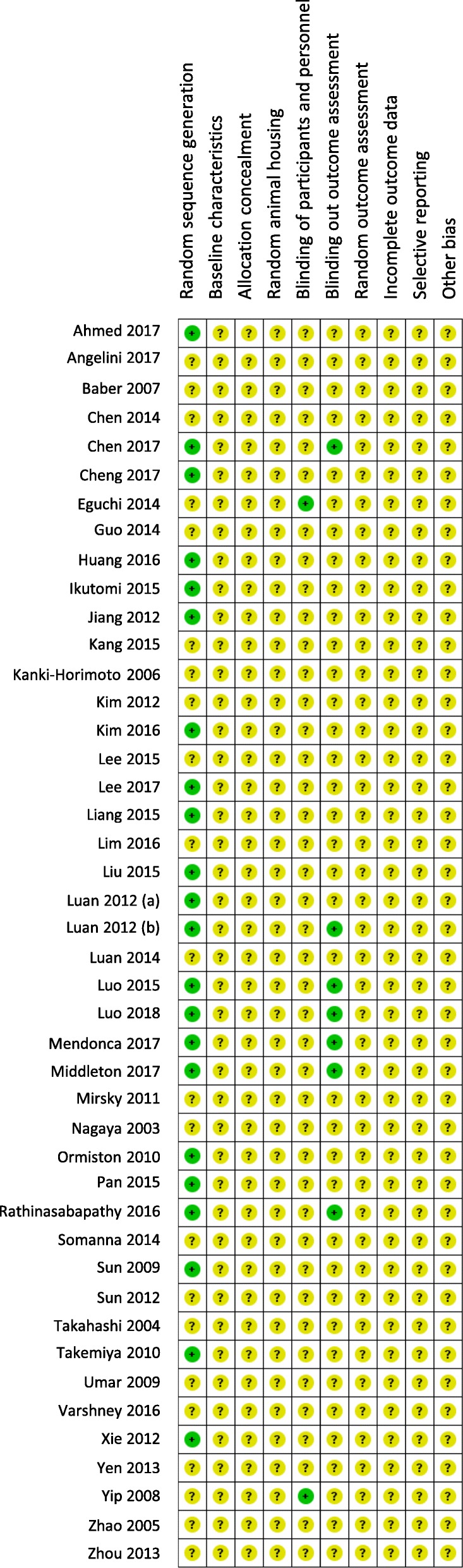
Risk of bias assessment using the SYRCLE tool

Most studies were rated for unclear risk of bias across all categories (Fig. 5), reflecting incomplete reporting of methodological details. Twenty-two studies (49%) described randomizing animals to treatment and control groups. Two studies (4%) reported blinding the experimenters, while seven studies (16%) reported blinding of outcome assessment. The risk of bias was unclear for all studies across the domains of allocation concealment, baseline characteristic description, random animal housing, random outcome assessment, incomplete outcome data, and selective reporting of outcomes.

## Discussion

Our systematic review is the first to examine the effect of regenerative cell therapy in preclinical models of PAH. Overall, the results of our meta-analysis demonstrate an improvement in the primary outcome hemodynamics (RVSP, mPAP) and the secondary outcome RV remodeling (RV/LV+S) with regenerative cell therapy. Subgroup analysis showed that the reduction in RVSP was observed in MSC, EPC, and iPSC cell types. Additional subgroup analysis on timing of treatment showed that regenerative cell therapy administered at 3, 7, 14, and 21 days post-induction resulted in significant reductions in RVSP. Efficacy was still observed when treatment was administered beyond 14 days post-induction during established PAH, which represents a clinically meaningful timepoint for intervention.

Several studies included in this meta-analysis examined the utility of cell enhancement strategies such as gene transfection or pharmacological preconditioning. Overall, cell enhancement was associated with greater reduction in RVSP and mPAP compared to treatment with non-enhanced cells. A caveat with this interpretation is that there is potential for publication bias, as ineffective enhancement strategies are less likely to be pursued and published. As well, due to the limited number of studies for each enhancement strategy, we could not compare between gene candidates or preconditioning.

All 45 studies were performed in the MCT model of PAH, and all performed in a male population of rats (*n* = 43 studies) or dogs (*n* = 2 studies) and therefore, the generalizability of these findings may be limited. The MCT model has been criticized for its off-target systemic toxicity to the liver, heart, and kidneys, which also limits the long-term study of treatments in preclinical studies. In recent years, the SU5416 + chronic hypoxia model of PAH has been considered the most representative model of human PAH due to its ability to closely replicate the salient histopathological features of PAH in humans and the profound changes in pulmonary hemodynamics [14, 21]. Ideally, confirmatory studies in both models would improve validity; however, our systematic search did not identify any studies involving regenerative cell therapy in the SU5416 + chronic hypoxia model.

One of the limitations of this study is that we did not assess histopathological data such as pulmonary vascular remodeling or cell engraftment. Regenerative cell therapy has been proposed to alter pulmonary vascular remodeling, perhaps by reducing endothelial apoptosis and limiting smooth muscle hyperplasia, and could therefore be a useful surrogate outcome to assess efficacy [8]. However, the lack of consensus scoring systems for assessing pulmonary vascular remodeling in vivo limited our ability to include this in the analysis. Indeed, metrics such as wall thickening, medial hypertrophy, and so forth have been reported, but the scoring criteria were often vague, subjective, and non standardized. As well, cell retention or engraftment in the pulmonary vasculature would support the vascular remodeling hypothesis of cell therapy. Similarly, we found that engraftment was measured by several methods including immunofluorescence staining, transfection with fluorescent tag, or PCR. Within the same study, different techniques (e.g., immunofluorescence vs PCR) yielded variable cell retention rates [22], likely due to differences in sensitivity and specificity for each technique.

Another limitation of this analysis is that functional outcome measurements were not available. In clinical trials, the 6-min walk test is widely used, [23]; however, no such standardized functional assessment exists for animal studies. Instead, other surrogate measures of RV function may be available. Indeed, RV function is arguably the most important predictor of prognosis in PAH [24]. However, accurate quantification of RV ejection fraction in small animals due to the unique and variable geometry of the RV is challenging [14, 23]. RV dysfunction precedes mortality, and therefore, assessment of RV function could be a useful tool for clinical translation. Tricuspid annular planar systolic excursion (TAPSE) and RV fractional area change are 2D echocardiography measures that were rarely reported in preclinical studies [23, 25] and thus not included in the meta-analysis. Future studies examining the effect of regenerative cell therapy on cardiac output (CO), cardiac index, or other indices of RV function may provide further insight into the long-term effects of cell therapy. Reporting of important outcomes such as cardiac output and pulmonary vascular resistance (PVR) was almost non-existent in included studies, and therefore, the effect of regenerative cell therapy on these outcomes could not be assessed.

Overall, the studies included in this systematic review were scored as having an unclear or high risk of bias using the itemized SYRCLE Risk of Bias tool for preclinical studies. For instance, although most studies randomize treatment, further measures to minimize bias such as allocation concealment or method of randomization were not reported. This information is critical, as inadequate generation or concealment of allocation is associated with exaggerated effect sizes [26]. Similarly, lack of blinding of outcome assessment, in which study investigators are unaware of the intervention allocation, is also associated with overestimation of effect size [27]. Only eight of the included studies reported blinding of outcome assessment. The lack of reporting of key potential sources of bias is consistent with preclinical literature in general. Although several preclinical reporting guidelines have been proposed to enhance transparency and methodological quality in preclinical studies [28], uptake by authors and journals has not been widespread. This lack of transparency contributes to poor reproducibility from preclinical to clinical trials, which is already strained as only < 5% of basic science discoveries are eventually approved by health authorities [29]. The identification of poor methodological reporting, as well as a lack of reporting of important outcomes such as CO and PVR, has identified a knowledge gap and represents a potential next step for future studies in the field.

In conclusion, regenerative cell therapy is associated with improved pulmonary hemodynamics and RV remodeling across several subgroups. The strengths of this study include the large number of studies [30], comprehensive data collection on model and treatment methodology, and subgroup analysis by cell enhancement, cell type, and timing, which can be used to inform future preclinical studies and clinical trials. The timing of this review is highly relevant, as small clinical trials have been completed (NCT00257413, NCT00641836, NCT00469027) [10, 11, 31]. So far, based on limited short-term data, the results of completed clinical trials have shown relatively modest benefits [10] compared to the effect sizes reported in some preclinical literature [32, 33]. The effect sizes in our review may be influenced in part by potential sources of bias, which may be improved by increased methodological rigor which include randomization, allocation concealment, and blinding in future studies. Alternatively, the difference in effect size could be contributed by the relative homogeneity of animal models of PAH, for example the rodent monocrotaline model, compared to clinical presentations of PAH. Future studies to address the validity of preclinical studies should also include the use of more comprehensive assessments of cardiac function, particularly the RV functional capacity, as well as follow-up confirmatory studies in multiple animal models such as the SU5416 + hypoxia model [27]. Several variations in regenerative cell therapy methods were evaluated for heterogeneity such as cell type, enhancement, and timing of administration. However, at this time, limited data and lack of head-to-head comparisons preclude the suggestion of an optimal method of cell therapy.

## Additional files

Additional file 1: PRISMA Checklist. (DOCX 19 kb)

Additional file 2: Search strategy and PRESS review. (DOCX 34 kb)

Additional file 3: **Figure S1.** Post hoc analysis of RVSP using mean difference (MD). **Figure S2.** Post hoc analysis of RVSP subgrouped by cell compatibility. **Figure S3.** Funnel plot indicating possible publication bias for RVSP. Open circles are included studies and black circles represent imputed studies from post hoc trim and fill analysis. **Figure S4.** Post hoc analysis of RVSP subgrouped by cell origin (MSC studies only). **Figure S5.** Post hoc analysis of RVSP subgrouped by cell dose (MSC studies only). **Figure S6.** Post hoc analysis of mPAP subgrouped by cell compatibility. **Figure S7.** Funnel plot indicating possible publication bias for mPAP. Open circles are included studies and black circles represent imputed studies from post hoc trim and fill analysis. **Figure S8.** Post hoc analysis of mPAP subgrouped by cell origin (MSC studies only). **Figure S9.** Post hoc analysis of mPAP subgrouped by cell dose (MSC studies only). **Figure S10.** Risk ratio and accompanying 95% confidence intervals for the risk of mortality. (DOCX 389 kb)
