# Supplementary material for: Regenerative cell therapy for pulmonary arterial hypertension in animal models: a systematic review
Source: Stem Cell Res Ther. 2019 Mar 6;10:75. doi: 10.1186/s13287-019-1172-6 (PMC6404277; doi:10.1186/s13287-019-1172-6)
Supplement: Supplementary file 2 — Search strategy and PRESS review. (DOCX 34 kb) [file 13287_2019_1172_MOESM2_ESM.docx]

**Additional file 2. Search strategy and PRESS review**

Database: Embase Classic+Embase &lt;1947 to 2014 October 20&gt;, Ovid MEDLINE(R) In-Process

&amp; Other Non-Indexed Citations and Ovid MEDLINE(R) &lt;1946 to Present&gt;

Search Strategy:

--------------------------------------------------------------------------------

1 exp pulmonary hypertension/ (90710)

2 (pulmonary adj2 hypertens*).tw. (78519)

3 PAH.tw. (36316)

4 or/1-3 (135956)

5 monocrotaline/ (3102)

6 (monocrotalin* or MCT).tw. (11211)

7 5 or 6 (11617)

8 4 or 7 (144819)

9 exp stem cell/ (355970)

10 ((stem or progenitor* or mother or precursor*) adj1 cell$1).tw. (477565)

11 exp bone marrow cell/ (244093)

12 (bone marrow adj3 cell$1).tw. (126737)

13 (BMD-EPC or BMD-EPCs or BMDEPC or BMDEPCs).tw. (23)

14 (&quot;colony-forming&quot; adj (cell$1 or unit or units)).tw. (39082)

15 (CFU or CFUs).tw. (73386)

16 ((embryoid or embroid or embryonic) adj2 (body or bodies or cell$1)).tw. (69160)

17 (angioblast* or hemangioblast* or haemangioblast*).tw. (7119)

18 myoblast$1.tw. (23537)

19 ((haemogenic or hemogenic) adj2 (endothelial or endothelium)).tw. (333)

20 ((osteoprogenitor adj cell$1) or OPC or OPCs).tw. (9519)

21 (side adj population adj cell$1).tw. (889)

22 (circulating angiogenic adj (cell or cells)).tw. (320)

23 (CAC or CACs).tw. (9315)

24 IPS cell$1.tw. (4165)

25 hiPSC$1.tw. (1259)

26 common lymphoid progenitor$1.tw. (548)

27 ((&quot;Pre-B&quot; or &quot;Pro-B&quot;) adj1 (cell$1 or lymphocyte*)).tw. (8461)

28 ((&quot;B-cell&quot; or &quot;B-cells&quot; or B-lymphcyte$1 or B-lymphoid$1) adj1 (immatur* or precursor* or

progenitor* or transitional)).tw. (8446)

29 ((&quot;T-cell&quot; or &quot;T-cells&quot; or T-lymphcyte$1 or T-lymphoid$1) adj1 (immatur* or precursor* or

progenitor* or transitional)).tw. (5642)

30 (thymocyte* or T-lymphocyte*).tw. (212857)

31 ((mesenchymal adj3 (stem or stroma$1 or progenitor* or precursor*)) and cell$1).tw.

(59423)

32 (MSC or MSCs or ADMSC or ADMSCs or BM-MSC or BM-MSCs or BMD-MSC or BMD-

MSCs or BMDMSC or BMDMSCs).tw. (37339)

33 ((multipotent or multi-potent) adj3 (stroma$1 cell$1 or stem cell$1)).tw. (6839)

34 marrow stroma$1 cell$1.tw. (12867)

35 CFU-F$1.tw. (1419)

36 mesenchymal.tw. and exp Gene Therapy/ (1822)

37 exp stem cell transplantation/ (146293)

38 ((hematopoietic or hemato-poietic or HSC or HSCs) adj10 transplant*).tw. (47378)

39 ((autologous or auto-logous or auto) adj HCT).tw. (561)

40 autoHCT.tw. (64)

41 ((allogeneic or allo-geneic or homologous or homo-logous) adj HCT).tw. (1531)

42 ((PBSC or PBSCs) adj10 transplant*).tw. (2278)

43 PBSCT.tw. (2878)

44 ((autologous or auto-logous or auto) adj (PBSC or PSC)).tw. (475)

45 (autoPBSCT or autoPBSC or autoPSC).tw. (98)

46 CBSCT.tw. (70)

47 stem cell mobilization/ (8718)

48 ((hematopoietic or hemato-poietic or HSC or HSCs) adj10 mobili#ation).tw. (2575)

49 autotransplantation/ (27048)

50 (transplant* adj1 (autologous or auto-logous)).tw. (8268)

51 allotransplantation/ (32908)

52 (transplant* adj1 (homologous or homo-logous or allogeneic or allo-geneic or isogeneic or

iso-geneic or syngeneic)).tw. (11476)

53 (allotransplant* or allo-transplant* or allograft* or allo-graft* or homograft* or homo-graft* or

isograft* or iso-graft*).tw. (147364)

54 exp bone marrow transplantation/ (99362)

55 (bone marrow adj2 (graft* or transplant*)).tw. (71314)

56 ((cell or cells or cell-based or tissue$1 or tissue-based) adj (therapy or therapies or

transplant*)).tw. (135200)

57 or/9-56 (1392649)

58 8 and 57 (3177)

59 exp animal experiment/ or exp animal model/ or animal/ or exp invertebrate Chordata/ or

exp experimental animal/ or exp transgenic animal/ or exp male animal/ or exp female animal/ or

exp juvenile animal/ or vertebrate/ or exp fish/ or exp amphibia/ or exp reptile/ or exp bird/ or

mammal/ or exp hyrax/ or exp marsupial/ or exp monotremate/ or exp scandentia/ or placental

mammals/ or exp bat/ or exp carnivora/ or exp cetacea/ or exp edentata/ or exp elephant/ or exp

insectivora/ or exp lagomorph/ or exp rodent/ or exp sirenia/ or exp ungulate/ or primate/ or exp

prosimian/ or haplorhini/ or exp tarsiiform/ or simian/ or exp platyrrhini/ or catarrhini/ or exp

cercopithecidae/ or ape/ or exp hylobatidae/ or hominid/ or exp chimpanzee/ or exp gorilla/ or exp

orang utan/ (11385941)

60 (animal$1 or chordata or vertebrate* or fish$2 or amphibian* or amphibium* or reptile$1 or

bird$1 or mammal* or dog or dogs or canine$1 or cat or cats or hyrax* or marsupial* or

monotrem* or scandentia or bat or bats or carnivor* or cetacea or edentata* or elephant* or insect

or insects or insectivore or lagomorph* or rodent$2 or mouse or mice or murine or murinae or

muridae or rat or rats or pig or pigs or piglet$1 or swine or rabbit$1 or sheep$1 or goat$1 or

horse$1 or equus or cow or cows or cattle or calf or calves or bovine or sirenia or ungulate$1 or

primate$1 or prosimian* or haplorhini* or tarsiiform* or simian*or platyrrhini or catarrhini or

cercopithecidae or ape or apes or hylobatidae or hominid* or chimpanzee* or gorilla* or

orangutan* or monkey or monkeys or ape or apes).tw. (8751457)

61 (preclinic* or pre-clinic*).tw. (148181)

62 or/59-61 (12992218)

63 58 and 62 (1309)

64 63 use emczd (785)

65 Hypertension, Pulmonary/ (79762)

66 (pulmonary adj2 hypertens*).tw. (78519)

67 PAH.tw. (36316)

68 or/65-67 (126579)

69 Monocrotaline/ (3102)

70 (monocrotalin* or MCT).tw. (11211)

71 69 or 70 (11617)

72 68 or 71 (135453)

73 exp Stem Cells/ (355970)

74 ((stem or progenitor* or mother or precursor*) adj1 cell$1).tw. (477565)

75 (&quot;colony-forming&quot; adj (cell$1 or unit or units)).tw. (39082)

76 (CFU or CFUs).tw. (73386)

77 ((embryoid or embroid or embryonic) adj2 (body or bodies or cell$1)).tw. (69160)

78 (angioblast* or hemangioblast* or haemangioblast*).tw. (7119)

79 myoblast$1.tw. (23537)

80 ((haemogenic or hemogenic) adj2 (endothelial or endothelium)).tw. (333)

81 ((osteoprogenitor adj cell$1) or OPC or OPCs).tw. (9519)

82 (side adj population adj cell$1).tw. (889)

83 (circulating angiogenic adj cell$1).tw. (320)

84 (CAC or CACs).tw. (9315)

85 IPS cell$1.tw. (4165)

86 hiPSC$1.tw. (1259)

87 common lymphoid progenitor$1.tw. (548)

88 ((&quot;Pre-B&quot; or &quot;Pro-B&quot;) adj1 (cell$1 or lymphocyte*)).tw. (8461)

89 ((&quot;Pre-B&quot; or &quot;Pro-B&quot;) adj1 (cell$1 or lymphocyte*)).tw. (8461)

90 ((&quot;B-cell&quot; or &quot;B-cells&quot; or B-lymphcyte$1 or B-lymphoid$1) adj1 (immatur* or precursor* or

progenitor* or transitional)).tw. (8446)

91 (thymocyte* or T-lymphocyte*).tw. (212857)

92 ((mesenchymal adj3 (stem or stroma$1 or progenitor* or precursor*)) and cell$1).tw.

(59423)

93 (MSC or MSCs or ADMSC or ADMSCs or BM-MSC or BM-MSCs or BMD-MSC or BMD-

MSCs or BMDMSC or BMDMSCs).tw. (37339)

94 ((multipotent or multi-potent) adj3 (stroma$1 cell$1 or stem cell$1)).tw. (6839)

95 marrow stroma$1 cell$1.tw. (12867)

96 CFU-F$1.tw. (1419)

97 exp Mesoderm/cy (6383)

98 mesenchymal.tw. and exp Genetic Therapy/ (1822)

99 exp Bone Marrow Cells/ (244093)

100 (bone marrow adj3 cell$1).tw. (126737)

101 (BMD-EPC or BMD-EPCs or BMDEPC or BMDEPCs).tw. (23)

102 exp Stem Cell Transplantation/ (146293)

103 ((hematopoietic or hemato-poietic or HSC or HSCs) adj10 transplant*).tw. (47378)

104 ((autologous or auto-logous or auto) adj HCT).tw. (561)

105 autoHCT.tw. (64)

106 ((allogeneic or allo-geneic or homologous or homo-logous) adj HCT).tw. (1531)

107 ((PBSC or PBSCs) adj10 transplant*).tw. (2278)

108 PBSCT.tw. (2878)

109 ((autologous or auto-logous or auto) adj (PBSC or PSC)).tw. (475)

110 (autoPBSCT or autoPBSC or autoPSC).tw. (98)

111 CBSCT.tw. (70)

112 Hematopoietic Stem Cell Mobilization/ (8718)

113 ((hematopoietic or hemato-poietic or HSC or HSCs) adj10 mobili#ation).tw. (2575)

114 Transplantation, Autologous/ (71353)

115 (transplant* adj1 (autologous or auto-logous)).tw. (8268)

116 (autograft* or auto-graft* or autotransplant* or auto-transplant*).tw. (41912)

117 exp Transplantation, Homologous/ (112785)

118 (transplant* adj1 (homologous or homo-logous or allogeneic or allo-geneic or isogeneic or

iso-geneic or syngeneic)).tw. (11476)

119 (allotransplant* or allo-transplant* or allograft* or allo-graft* or homograft* or homo-graft* or

isograft* or iso-graft*).tw. (147364)

120 Bone Marrow Transplantation/ (86465)

121 (bone marrow adj2 (graft* or transplant*)).tw. (71314)

122 exp &quot;Cell- and Tissue-Based Therapy&quot;/ (1214515)

123 ((cell or cells or cell-based or tissue$1 or tissue-based) adj (therapy or therapies or

transplant*)).tw. (135200)

124 or/73-123 (2458396)

125 72 and 124 (6145)

126 exp animal experimentation/ or exp models, animal/ or animals/ or mammals/ or

vertebrates/ or exp fishes/ or exp amphibia/ or exp reptiles/ or exp birds/ or exp hyraxes/ or exp

marsupialia/ or exp monotremata/ or exp scandentia/ or exp chiroptera/ or exp carnivora/ or exp

cetacea/ or exp Xenarthra/ or exp elephants/ or exp insectivora/ or exp lagomorpha/ or exp

rodentia/ or exp sirenia/ or exp Perissodactyla/ or primates/ or exp strepsirhini/ or haplorhini/ or

exp tarsii/ or exp platyrrhini/ or catarrhini/ or exp cercopithecidae/ or gorilla gorilla/ or pan

paniscus/ or pan troglodytes/ or exp pongo/ or exp hylobatidae/ or hominidae/ (10980216)

127 (animal$1 or chordata or vertebrate* or fish$2 or amphibian* or amphibium* or reptile$1 or

bird$1 or mammal* or dog or dogs or canine$1 or cat or cats or hyrax* or marsupial* or

monotrem* or scandentia or bat or bats or carnivor* or cetacea or edentata* or elephant* or insect

or insects or insectivore or lagomorph* or rodent$2 or mouse or mice or murine or murinae or

muridae or rat or rats or pig or pigs or piglet$1 or swine or rabbit$1 or sheep$1 or goat$1 or

horse$1 or equus or cow or cows or cattle or calf or calves or bovine or sirenia or ungulate$1 or

primate$1 or prosimian* or haplorhini* or tarsiiform* or simian*or platyrrhini or catarrhini or

cercopithecidae or ape or apes or hylobatidae or hominid* or chimpanzee* or gorilla* or

orangutan* or monkey or monkeys or ape or apes).tw. (8751457)

128 exp Drug Evaluation, Preclinical/ (315539)

129 (preclinic* or pre-clinic*).tw. (148181)

130 or/126-129 (12788789)

131 125 and 130 (1867)

132 131 use prmz (577)

133 64 or 132 (1362)

134 remove duplicates from 133 (955)

135 134 use prmz (528)

136 134 use emczd (427)

# PRESS EBC Search Submission

**ESS**

**Searcher’s Name: Becky Skidmore / Colin Suen** **E-mail:** [bskidmore@rogers.com](mailto:bskidmore@rogers.com) **;** [cmsuen@gmail.com](mailto:cmsuen@gmail.com)

**Date submitted: 16 Oct 2014 Date needed by: ASAP**

***Note to peer reviewers – please enter your information in the Peer Review Assessment area***

Remember: this peer review only pertains to your MEDLINE search strategy.

***Search question*** (Describe the purpose of the search)

In preclinical studies of pulmonary arterial hypertension, does stem/progenitor cell-based therapy improve the severity of PAH?

***PICO format*** (Outline the PICO for your question, i.e., the Patient, Intervention, Comparison and Outcome)

***P:*** Preclinical *in vivo* models of pulmonary hypertension

***I:*** ANY stem or progenitor cell (xenogenic; syn/allogenic) infusion following the induction of PAH

***C:*** Animals with pulmonary hypertension not receiving stem cell therapy

***O:*** Pulmonary hemodynamics, right ventricular remodeling and death

***Inclusion criteria*** (List any inclusion criteria, such as age groups, study designs, to be included)

Any animal/pre-clinical study

***Exclusion criteria*** (List any exclusion criteria, such as study designs, to be excluded)

Neonatal

In vitro studies

Human studies

LVEDP >20 or LVEF<50 (excludes left heart failure)

Models of valvulopathy (mitral regurgitation/stenosis)

Models of pulmonary embolism; or models of malignancy with associated PH

Models of lung diseases such as idiopathic pulmonary fibrosis, emphysema etc,

Genetically modified animal models

***Was a search filter applied?*** (Remember this pertains only to the MEDLINE strategy)

| Yes |  | No |  |  |  |  |  |
| --- | --- | --- | --- | --- | --- | --- | --- |

***If yes, which one?***

Cochrane hedge: PUBMED clinical query:

Haynes/McKibbon et al: SIGN (Scottish):

CRD (UK): Robinson and Dickerson:

Other: *Animal/Pre-Clinical Filter – Based on*:

Enhancing search efficiency by means of a search filter for finding all studies on animal experimentation in PubMed

<http://www.ncbi.nlm.nih.gov/pubmed/20551243>

full text: <http://www.ncbi.nlm.nih.gov/pmc/articles/PMC3104815/>

***MEDLINE search interface used***

| *EBSCO* |  | OVID |  | *PubMED* |  | *Other* | ___________________ |
| --- | --- | --- | --- | --- | --- | --- | --- |

Has the search strategy been adapted (i.e., subject heading and terms reviewed) for other databases? Please check all that apply.

| Ageline | |  | |
| --- | --- | --- | --- |
| AMED | |  | |
| C2-SPCTRE | |  | |
| CINAHL | |  | |
| [Cochrane Database of Systematic Reviews (CDSR; Cochrane Reviews)](http://www3.interscience.wiley.com/cgi-bin/mrwhome/106568753/ProductDescriptions.html#creviews#creviews) | |  | |
| [Cochrane Central Register of Controlled Trials (CENTRAL; Clinical Trials)](http://www3.interscience.wiley.com/cgi-bin/mrwhome/106568753/ProductDescriptions.html#central#central) | |  | |
| [Cochrane Methodology Register (CMR; Methods Studies)](http://www3.interscience.wiley.com/cgi-bin/mrwhome/106568753/ProductDescriptions.html#cmr#cmr) | |  | |
| Cochrane Library (all databases) | |  | |
| [Database of Abstracts of Reviews of Effects (DARE; Other Reviews)](http://www3.interscience.wiley.com/cgi-bin/mrwhome/106568753/ProductDescriptions.html#dare#dare) | |  | |
| Embase | |  | |
| ERIC | |  | |
| LILACS (Latin American and Caribbean Health Sciences Literature) | |  | |
| MEDLINE | |  | |
| PsycINFO | |  | |
| PreMEDLINE | |  | |
| Other | |  | |
| Other | |  | |
| Other | |  | |

***Other notes or comments that you feel would be useful for the peer reviewer?***

*Did not search Cochrane as not relevant to animal/pre-clinical studies*

*Monocrotaline used to induce PAH – separated out in strategy for visual purposes only*

***Please paste your MEDLINE strategy here:***

Database: Ovid MEDLINE(R) In-Process & Other Non-Indexed Citations and Ovid MEDLINE(R) <1946 to Present> Search Strategy:

--------------------------------------------------------------------------------

1 Hypertension, Pulmonary/ (25805)

2 (pulmonary adj2 hypertens*).tw. (31812)

3 PAH.tw. (14739)

4 or/1-3 (49193)

5 Monocrotaline/ (1121)

6 (monocrotalin* or MCT).tw. (4849)

7 5 or 6 (4935)

8 4 or 7 (52984)

9 exp Stem Cells/ (148753)

10 ((stem or progenitor* or mother or precursor*) adj1 cell$1).tw. (210662)

11 ("colony-forming" adj (cell$1 or unit or units)).tw. (18481)

12 (CFU or CFUs).tw. (34253)

13 ((embryoid or embroid or embryonic) adj2 (body or bodies or cell$1)).tw. (32803)

14 (angioblast* or hemangioblast* or haemangioblast*).tw. (3114)

15 myoblast$1.tw. (11153)

16 ((haemogenic or hemogenic) adj2 (endothelial or endothelium)).tw. (148)

17 ((osteoprogenitor adj cell$1) or OPC or OPCs).tw. (3714)

18 (side adj population adj cell$1).tw. (401)

19 (circulating angiogenic adj cell$1).tw. (125)

20 (CAC or CACs).tw. (3844)

21 IPS cell$1.tw. (1698)

22 hiPSC$1.tw. (581)

23 common lymphoid progenitor$1.tw. (245)

24 (("Pre-B" or "Pro-B") adj1 (cell$1 or lymphocyte*)).tw. (4045)

25 (("B-cell" or "B-cells" or B-lymphcyte$1 or B-lymphoid$1) adj1 (immatur* or precursor* or progenitor* or transitional)).tw. (3807)

26 (("T-cell" or "T-cells" or T-lymphcyte$1 or T-lymphoid$1) adj1 (immatur* or precursor* or progenitor* or transitional)).tw. (2715)

27 (thymocyte* or T-lymphocyte*).tw. (100019)

28 ((mesenchymal adj3 (stem or stroma$1 or progenitor* or precursor*)) and cell$1).tw. (25008)

29 (MSC or MSCs or ADMSC or ADMSCs or BM-MSC or BM-MSCs or BMD-MSC or BMD-MSCs or BMDMSC or BMDMSCs).tw. (15568)

30 ((multipotent or multi-potent) adj3 (stroma$1 cell$1 or stem cell$1)).tw. (3136)

31 marrow stroma$1 cell$1.tw. (5795)

32 CFU-F$1.tw. (598)

33 exp Mesoderm/cy [Cytology] (6383)

34 mesenchymal.tw. and exp Genetic Therapy/ (623)

35 exp Bone Marrow Cells/ (159688)

36 (bone marrow adj3 cell$1).tw. (54898)

37 (BMD-EPC or BMD-EPCs or BMDEPC or BMDEPCs).tw. (7)

38 exp Stem Cell Transplantation/ (57635)

39 ((hematopoietic or hemato-poietic or HSC or HSCs) adj10 transplant*).tw. (19206)

40 ((autologous or auto-logous or auto) adj HCT).tw. (196)

41 autoHCT.tw. (13)

42 ((allogeneic or allo-geneic or homologous or homo-logous) adj HCT).tw. (535)

43 ((PBSC or PBSCs) adj10 transplant*).tw. (931)

44 PBSCT.tw. (1223)

45 ((autologous or auto-logous or auto) adj (PBSC or PSC)).tw. (185)

46 (autoPBSCT or autoPBSC or autoPSC).tw. (35)

47 CBSCT.tw. (32)

48 Hematopoietic Stem Cell Mobilization/ (3546)

49 ((hematopoietic or hemato-poietic or HSC or HSCs) adj10 mobili#ation).tw. (1050)

50 Transplantation, Autologous/ (44305)

51 (transplant* adj1 (autologous or auto-logous)).tw. (3415)

52 (autograft* or auto-graft* or autotransplant* or auto-transplant*).tw. (18253)

53 exp Transplantation, Homologous/ (79877)

54 (transplant* adj1 (homologous or homo-logous or allogeneic or allo-geneic or isogeneic or iso-geneic or syngeneic)).tw. (4655)

55 (allotransplant* or allo-transplant* or allograft* or allo-graft* or homograft* or homo-graft* or isograft* or iso-graft*).tw. (62848)

56 Bone Marrow Transplantation/ (42434)

57 (bone marrow adj2 (graft* or transplant*)).tw. (32341)

58 exp "Cell- and Tissue-Based Therapy"/ (12176)

59 ((cell or cells or cell-based or tissue$1 or tissue-based) adj (therapy or therapies or transplant*)).tw. (54527)

60 or/9-59 (709389)

61 8 and 60 (1159)

62 exp animal experimentation/ or exp models, animal/ or animals/ or mammals/ or vertebrates/ or exp fishes/ or exp amphibia/ or exp reptiles/ or exp birds/ or exp hyraxes/ or exp marsupialia/ or exp monotremata/ or exp scandentia/ or exp chiroptera/ or exp carnivora/ or exp cetacea/ or exp Xenarthra/ or exp elephants/ or exp insectivora/ or exp lagomorpha/ or exp rodentia/ or exp sirenia/ or exp Perissodactyla/ or primates/ or exp strepsirhini/ or haplorhini/ or exp tarsii/ or exp platyrrhini/ or catarrhini/ or exp cercopithecidae/ or gorilla gorilla/ or pan paniscus/ or pan troglodytes/ or exp pongo/ or exp hylobatidae/ or hominidae/ (5601001)

63 (animal$1 or chordata or vertebrate* or fish$2 or amphibian* or amphibium* or reptile$1 or bird$1 or mammal* or dog or dogs or canine$1 or cat or cats or hyrax* or marsupial* or monotrem* or scandentia or bat or bats or carnivor* or cetacea or edentata* or elephant* or insect or insects or insectivore or lagomorph* or rodent$2 or mouse or mice or murine or murinae or muridae or rat or rats or pig or pigs or piglet$1 or swine or rabbit$1 or sheep$1 or goat$1 or horse$1 or equus or cow or cows or cattle or calf or calves or bovine or sirenia or ungulate$1 or primate$1 or prosimian* or haplorhini* or tarsiiform* or simian*or platyrrhini or catarrhini or cercopithecidae or ape or apes or hylobatidae or hominid* or chimpanzee* or gorilla* or orangutan* or monkey or monkeys or ape or apes).tw. (3982758)

64 exp Drug Evaluation, Preclinical/ (175932)

65 (preclinic* or pre-clinic*).tw. (63734)

66 or/62-65 (6289628)

67 61 and 66 (576)

***************************

**Peer Review Assessment**

**[For peer reviewers only]**

**Peer reviewer’s name:**

**Press #:**

**E-mail:**

**Date completed:**

### Please select the one most appropriate answer for each element

|  | **Adequate** | **Adequate with revisions*** | **Needs revision*** |
| --- | --- | --- | --- |
| 1. Translation of the research question | X |  |  |
| 2. Boolean and proximity operators | x |  |  |
| 3. Subject headings | X |  |  |
| 4. Natural language / free-text | X |  |  |
| 5. Spelling, syntax and line numbers | X |  |  |
| 6. Limits and filters | X |  |  |
| 7. Search strategy adaptations | X |  |  |

*** Provide an explanation or example for “Adequate with revisions” and “needs revision”:

Other Comments (please limit to 3-5 sentences):

Very thorough.
