# Supplementary material for: Regenerative cell therapy for pulmonary arterial hypertension in animal models: a systematic review
Source: Stem Cell Res Ther. 2019 Mar 6;10:75. doi: 10.1186/s13287-019-1172-6 (PMC6404277; doi:10.1186/s13287-019-1172-6)

**Additional file 3**

**Figure S1.** Post-hoc analysis of RVSP using mean difference (MD)


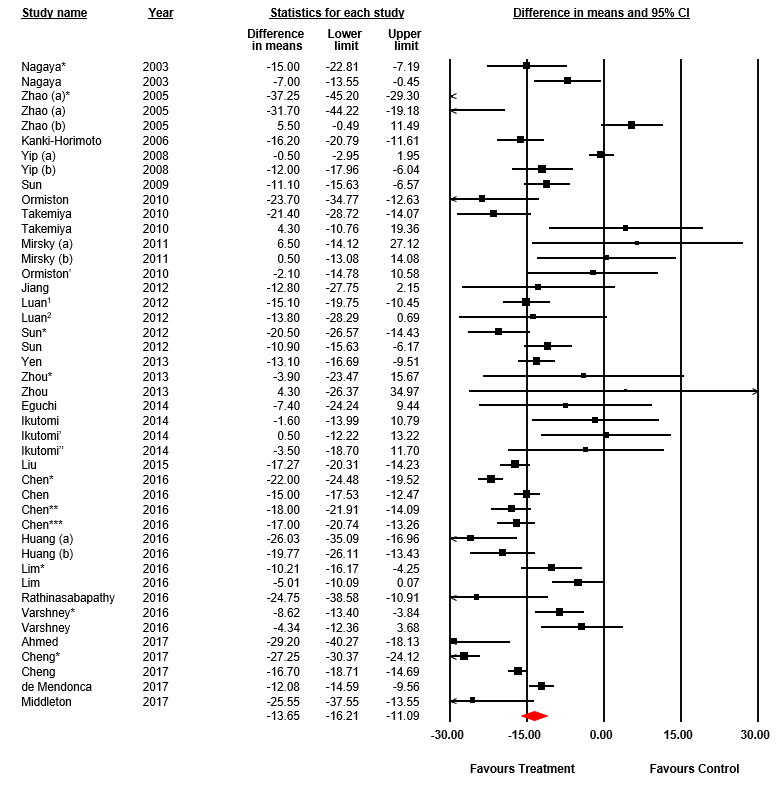


**Figure S2.** Post-hoc analysis of RVSP subgrouped by cell compatibility


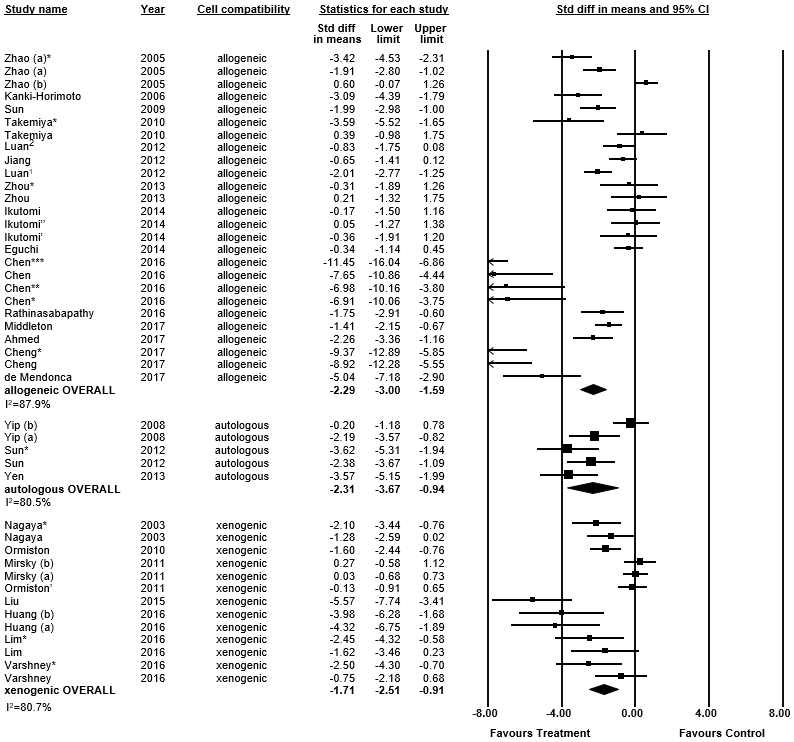


**Figure S3.** Funnel plot indicating possible publication bias for RVSP. Open circles are included studies and black circles represent imputed studies from post-hoc trim and fill analysis.

**Figure S4.** Post-hoc analysis of RVSP subgrouped by cell origin (MSC studies only)


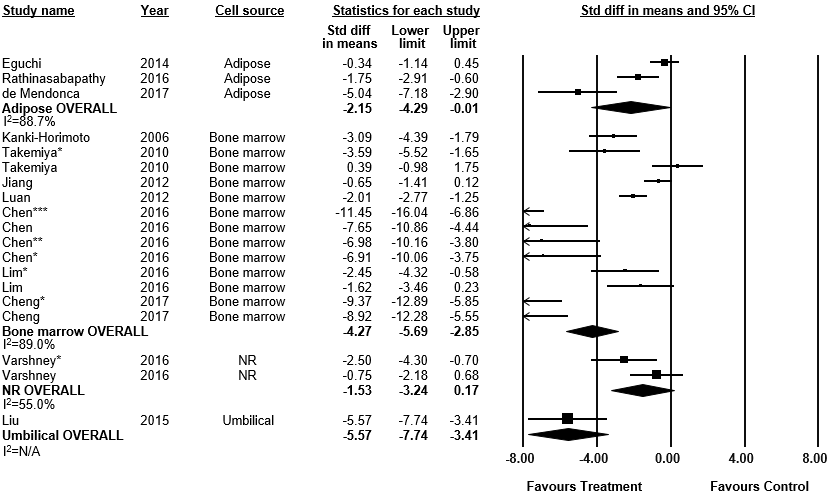


**Figure S5.** Post-hoc analysis of RVSP subgrouped by cell dose (MSC studies only)


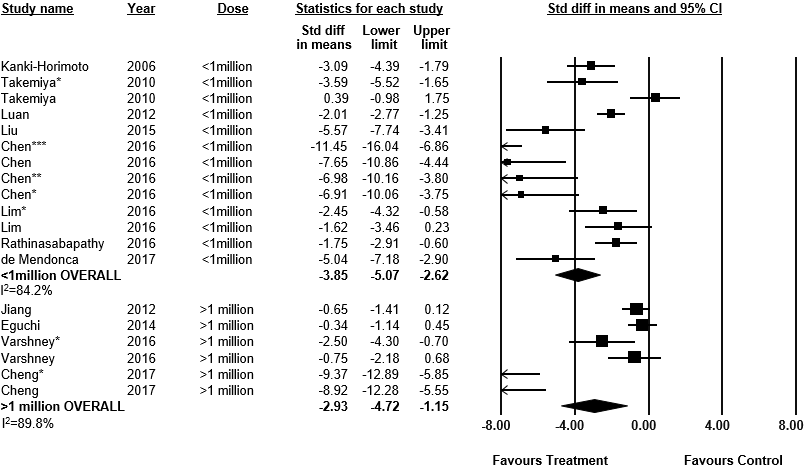


**Figure S6.** Post-hoc analysis of mPAP subgrouped by cell compatibility


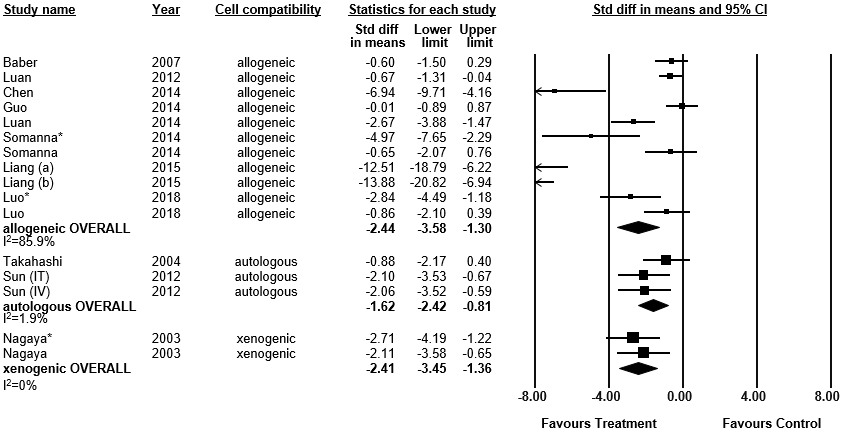


**Figure S7.** Funnel plot indicating possible publication bias for mPAP. Open circles are included studies and black circles represent imputed studies from post-hoc trim and fill analysis.

**Figure S8.** Post-hoc analysis of mPAP subgrouped by cell origin (MSC studies only)


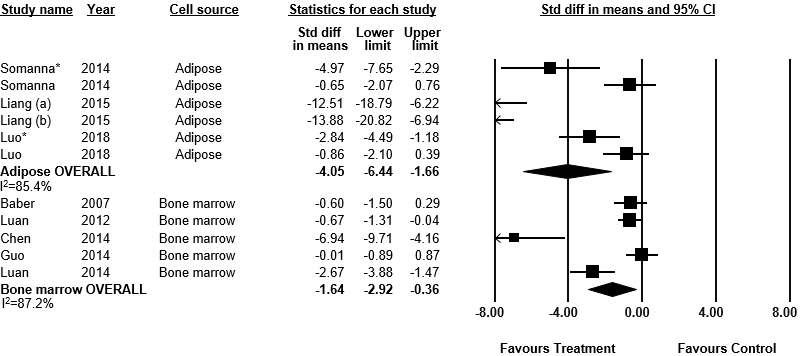


**Figure S9.** Post-hoc analysis of mPAP subgrouped by cell dose (MSC studies only)


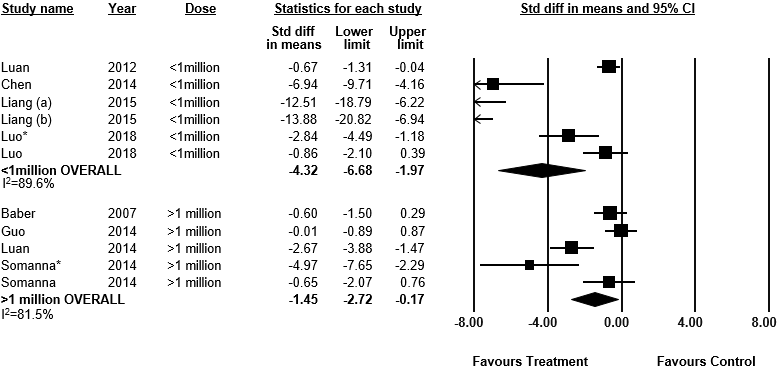


**Figure S10.** Risk ratio and accompanying 95% confidence intervals for the risk of mortality


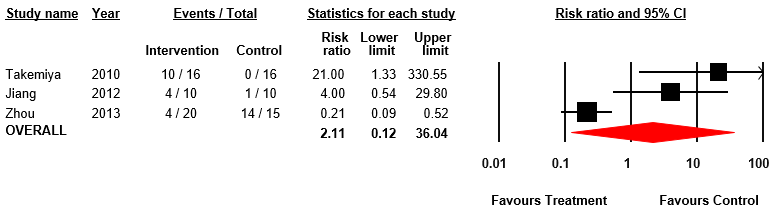

Supplement: Supplementary file 3 — Figure S1. Post hoc analysis of RVSP using mean difference (MD). Figure S2. Post hoc analysis of RVSP subgrouped by cell compatibility. Figure S3. Funnel plot indicating possible publication bias for RVSP. Open circles are included studies and black circles represent imputed studies from post hoc trim and fill analysis. Figure S4. Post hoc analysis of RVSP subgrouped by cell origin (MSC studies only). Figure S5. Post hoc analysis of RVSP subgrouped by cell dose (MSC studies only). Figure S6. Post hoc analysis of mPAP subgrouped by cell compatibility. Figure S7. Funnel plot indicating possible publication bias for mPAP. Open circles are included studies and black circles represent imputed studies from post hoc trim and fill analysis. Figure S8. Post hoc analysis of mPAP subgrouped by cell origin (MSC studies only). Figure S9. Post hoc analysis of mPAP subgrouped by cell dose (MSC studies only). Figure S10. Risk ratio and accompanying 95% confidence intervals for the risk of mortality. (DOCX 389 kb) [file 13287_2019_1172_MOESM3_ESM.docx]
